# Measles, Mumps, Rubella and Varicella Among Service Members and Other Beneficiaries of the Military Health System, 2019–2024

**Published:** 2025-10-01

**Authors:** Sithembile L. Mabila, Michael T. Fan, Shauna L. Stahlman

**Affiliations:** Epidemiology and Analysis Branch, Armed Forces Health Surveillance Division, Public Health Directorate, Defense Health Agency, Silver Spring, MD

## Abstract

Measles, mumps, rubella, and varicella (MMR/V) cases have decreased in the U.S. Military Health System (MHS) overall, but in recent years, increasing numbers of MMR/V outbreaks in the U.S. have led to a rise in reported cases among the civilian population. Data were queried from the Defense Medical Surveillance System to identify total number of confirmed and possible MMR/V cases among all MHS beneficiaries from 2019 through 2024. The total numbers of confirmed and possible cases among MHS beneficiaries included 8 confirmed and 71 possible cases of measles, 18 confirmed and 193 possible cases of mumps, 13 confirmed and 265 possible cases of rubella, and 251 confirmed and 4,554 possible cases of varicella. During the surveillance period the numbers of all confirmed and possible cases decreased. Among service members, most cases were either partially vaccinated, or vaccination records were not available.

Although the numbers of measles, mumps, rubella, and varicella (MMR/V) cases have drastically declined in the U.S. after vaccine implementation, outbreaks of these diseases still occur sporadically. ^1 , 2^ Fourteen measles outbreaks occurred in the U.S. between January 1 and May 8, 2025, accounting for 1,001 confirmed measles cases reported by 31 U.S. jurisdictions, 126 (12.6%) hospitalizations, and 3 deaths. Mumps outbreaks also continue to occur across the U.S., with cases drastically increasing in 2016 (n=6,366 cases) compared to the previous 5 years, during which cases ranged from 200 to 1,329 annually. ^4^ Even though the number of total cases of mumps has decreased since 2016, with cases dropping below 500 cases per year, from 2021 through 2025, mumps cases are still reported annually, with 357 cases reported in 2024. ^4^ Varicella cases have also drastically decreased since the introduction of the 2-dose vaccine in 2007, from an average rate of 215 cases per 100,000 population, 1994–1995, to 33 cases per 100,000 population. ^5^ The median number of rubella cases reported annually, 2001–2004, was 14 (range 7-23), and rubella was declared eliminated in the U.S. in 2004. ^6^ Rubella is no longer endemic to the U.S., with its annual 2005–2022 incidence remaining less than 1 case per 10 million population, with most reported cases in the recent past acquired while traveling or living outside the U.S. ^6^ It remains important to monitor MMR/V cases in the U.S. Military Health System (MHS), as service members deploy to other countries where MMR/V is endemic, and viral outbreaks continue to occur within the U.S.

The Standing Order for Administering MMR/V vaccine among adults outlines the U.S. Department of Defense (DOD) policy for MMR/V vaccination. ^7^ Military environments such as recruit training locations, barracks, and ships are conducive to the spread of MMR/V because service members live in close quarters. Military personnel are required to receive the MMR/V vaccine and provide documentation of 2 lifetime doses of MMR/V-containing vaccines, or serological evidence of immunity. If no documentation is available, 1 dose of MMR/V-containing vaccine is administered within the first 2 weeks of initial training, and the second dose is administered at least 4 weeks later. *MSMR* has previously reported on MMR/V cases among MHS beneficiaries, describing trends from 2010 through 2016 and 2016 through 2019. ^8 , 9^ From 2016 through June 2019, the total number of MMR/V cases were relatively low among MHS beneficiaries, with 5 confirmed cases of measles and 64 confirmed cases of mumps. None of the measles cases were among service members. ^9^

**What are the new findings?**

In this 6-year surveillance period, cases of MMR/V decreased over time. No cases of measles were observed among U.S. service members during the surveillance period.

**What is the impact on readiness and force health protection?**

This report emphasizes the importance of continued vaccination against MMR/V to limit morbidity among U.S. service members, as evidenced by the lower number of cases among service members, who are required to be vaccinated, when compared to non-service members.

This analysis provides an update on MMR/V cases from 2019 through 2024 to describe temporal trends among MHS beneficiaries. Additionally, this analysis stratifies cases by MMR/V immunization status to evaluate waning immunity and breakthrough infections among service members.

## Methods

This retrospective cohort study included all MHS beneficiaries from 2019 through 2024. Demographic, immunization, and medical encounter data were obtained from the Defense Medical Surveillance System (DMSS). Because MMR/V are considered reportable medical events (RMEs), RME data for confirmed and possible cases were evaluated, in addition to International Classification of Diseases, 9th and 10th Revisions, Clinical Modification (ICD-9/10-CM) diagnostic codes from medical encounter data.

The Armed Forces Health Surveillance Division surveillance case definitions for MMR/V were used for this analysis. In summary, a ‘confirmed’ case was defined as an individual identified through an RME of MMR/V that was described as confirmed according to laboratory and epidemiological criteria. ^10 - 13^ A ‘possible’ case was defined as 1) a suspect, probable, unknown, or pending RME of MMR/V or 2) a record of an inpatient or outpatient medical encounter with a diagnosis of measles, mumps, rubella, or varicella in the primary diagnostic position.

For measles, mumps, and rubella cases, a disease-associated symptom in any other diagnosis position was also required in addition to the aforementioned RME or medical encounter requirement for possible cases. ^10 - 13^ Encounters with a record of MMR/V immunization or positive test for serological immunity to MMR/V within 7 days of the encounter date, or an ICD-10-CM diagnosis or a Current Procedural Terminology (CPT) code indicating MMR/V vaccination on the same day as the MMR/V diagnosis were excluded. ^10 - 13^

Vaccination status for service member cases was determined using the immunization data from the immunization table in DMSS. Immunization types for measles (03, 04, 05, 94), mumps (03, 07, 038, 94), rubella (03, 04, 06, 38, 94) and varicella (21, 36, 117, 94) were queried. A fully vaccinated case was an individual who had received 2 MMR/V vaccine doses at least 28 days apart, while any cases with 1 dose were considered partially vaccinated. Individuals without any vaccination information, or those with vaccination information after an incident case, were considered unvaccinated. Immunization exemption data were queried to determine cases that were exempt from the MMR/V vaccine. MHS beneficiaries were stratified by component and service. Due to the limited number of cases among service members, incident rates and any further analysis were not performed. The immunization table in DMSS does not have immunization data for non-service members; thus, the vaccination status of non-service members was not determined. All analyses were conducted using SAS-Enterprise Guide (version 8.3).

## Results

### Measles

**FIGURE 1.**
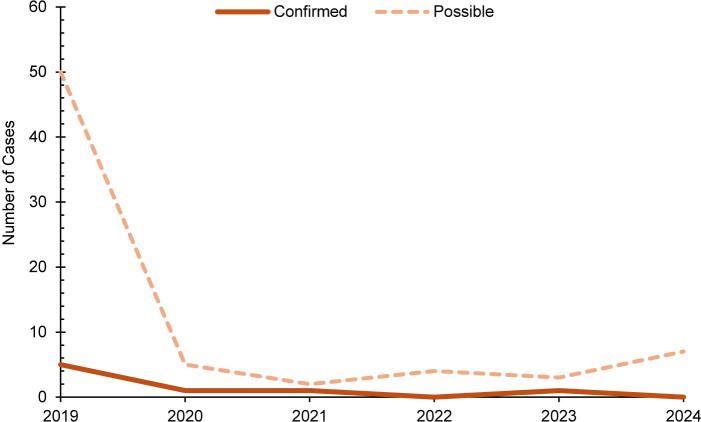
Annual Measles Cases, All Military Health System Beneficiaries, 2019–2024

**FIGURE 2.**
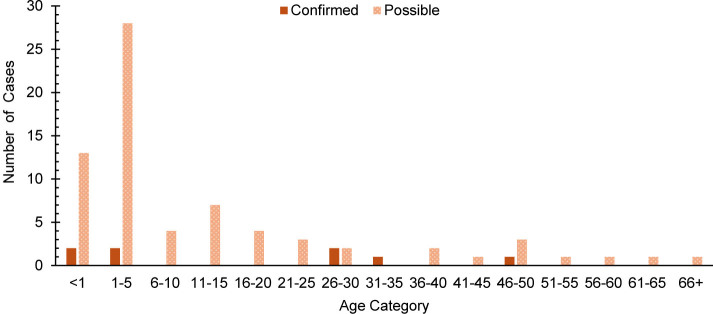
Age Distribution of Confirmed and Possible Measles Cases, All Military Health System Beneficiaries, 2019–2024

This retrospective study identified a total of 8 confirmed and 71 possible cases of measles among all MHS beneficiaries during the surveillance period **(Table 1)** . No confirmed measles cases were among U.S. service members. Of the 71 possible measles cases, the majority (n=69, 97.2%) were among non-service member beneficiaries. Overall, both confirmed and possible cases of measles decreased during the surveillance period **(Figure 1)** . Half of confirmed measles cases (n=4, 50.0%) and over half of possible measles cases (n=41, 57.7%) were among children ages 5 years or younger **(Figure 2)** .

**TABLE 1.** Confirmed and Possible Cases of Measles, Mumps, Rubella and Varicella, All Military Health System Beneficiaries, 2019–2024

	Measles		Mumps		Rubella		Varicella	
	Confirmed	Possible	Confirmed	Possible	Confirmed	Possible	Confirmed	Possible
	No.	No.	No.	No.	No.	No.	No.	No.
Total	8	71	18	193	13	265	251	4,554
Component								
Active component	0	1	7	61	6	22	68	359
Reserve component, National Guard	0	1	2	2	0	2	4	124
Non-service member beneficiaries	8	69	9	130	7	241	179	4,071
Sex								
Male	5	38	13	117	4	123	139	2,158
Female	3	33	5	76	9	142	112	2,396
Service branch ^a^								
Army	0	0	3	33	2	9	18	198
Navy	0	1	5	12	3	6	24	85
Air Force	0	1	1	6	1	6	21	132
Marine Corps	0	0	0	11	0	3	8	51
Space Force	0	0	0	0	0	0	1	2
Coast Guard	0	0	0	1	0	0	0	15

Abbreviation: No., number.

a Among active component, reserve component, and National Guard service members.

### Mumps

**FIGURE 3.**
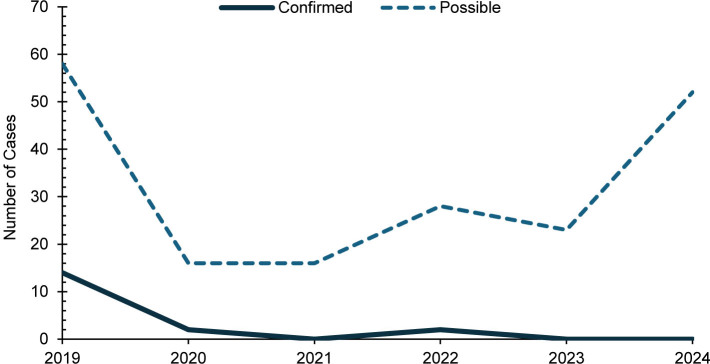
Annual Mumps Cases, All Military Health System Beneficiaries, 2019–2024

**FIGURE 4.**
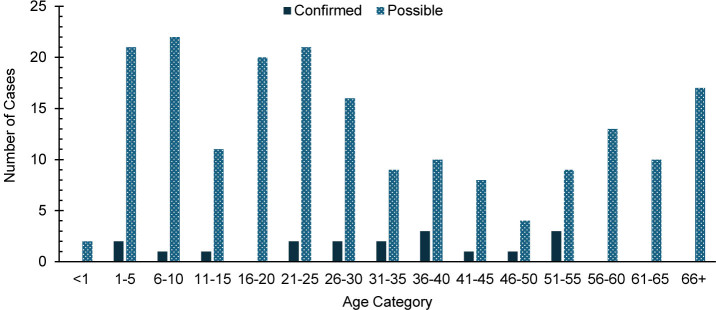
Age Distribution of Confirmed and Possible Mumps Cases, All Military Health System Beneficiaries, 2019–2024

A total of 18 confirmed and 193 possible mumps cases were identified among all MHS beneficiaries during the surveillance period. Half of confirmed mumps cases (n=9) occurred among service members. Among the 193 possible cases, a majority (n=130, 67.4%) were among non-service member beneficiaries **(Table 1)** . The greatest annual number of confirmed cases (n=14) for all MHS beneficiaries occurred in 2019 **(Figure 3)** . Cases were sporadically distributed among age categories **(Figure 4)** . Of the 9 confirmed mumps cases among service members, 4 had been fully vaccinated, 2 partially vaccinated, and 3 had not been vaccinated **(Table 2)** .

**TABLE 2.** Vaccination Status of Confirmed Mumps, Rubella and Varicella Cases, U.S. Service Members, by Year, 2019–2024

	Status of Vaccination	2019	2020	2021	2022	2023	2024	Total
		No.	No.	No.	No.	No.	No.	No.
Mumps	Fully vaccinated	3	1	0	0	0	0	4
	Partially vaccinated	2	0	0	0	0	0	2
	Not vaccinated	2	1	0	0	0	0	3
	Exempt	0	0	0	0	0	0	0
Rubella	Fully vaccinated	0	0	0	0	0	0	0
	Partially vaccinated	0	0	1	1	1	0	3
	Not vaccinated	0	0	0	0	0	0	0
	Exempt	0	0	1	1	1	0	3
Varicella	Fully vaccinated	0	1	1	3	0	2	7
	Partially vaccinated	0	2	1	1	1	0	5
	Not vaccinated	3	2	0	4	1	2	12
	Exempt	11	7	11	7	7	5	48

Abbreviation: No., number.

### Rubella

**FIGURE 5.**
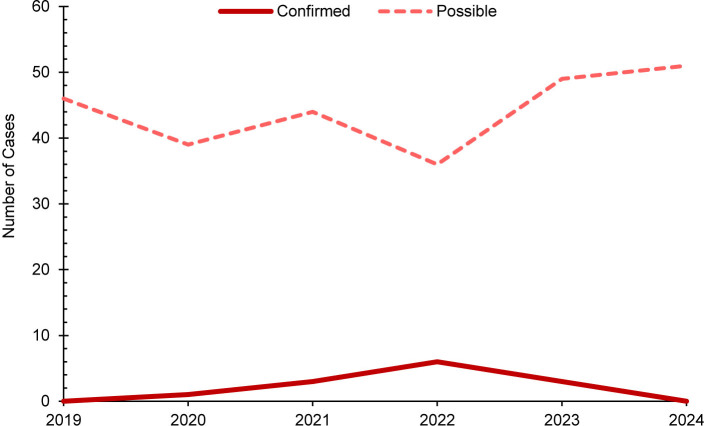
Annual Rubella Cases, All Military Health System Beneficiaries, 2019–2024

**FIGURE 6.**
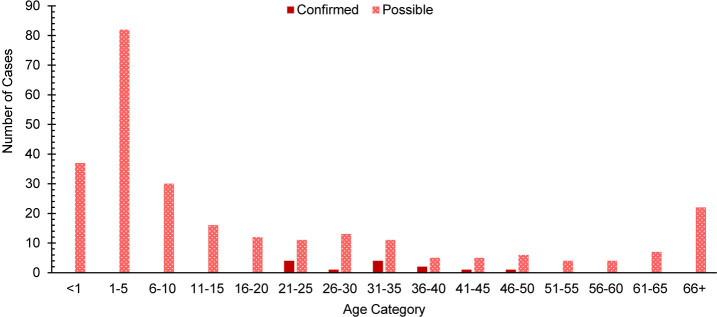
Age Distribution of Confirmed and Possible Rubella Cases, All Military Health System Beneficiaries, 2019–2024

A total of 13 confirmed and 265 possible rubella cases were identified among all MHS beneficiaries during the surveillance period. Six of the confirmed rubella cases occurred among active component service members. Among the 265 possible cases, a majority (n=241, 90.9%) were among non-service member beneficiaries **(Table 1)** . Confirmed rubella cases peaked in 2022 (n=6), subsequently declining to 0 cases in 2024 **(Figure 5)** . All confirmed rubella cases were among those aged 21 years and older **(Figure 6)** . Among the confirmed service member cases, 3 had been partially vaccinated, and 3 cases had received an exemption from vaccination **(Table 2)** .

### Varicella

**FIGURE 7.**
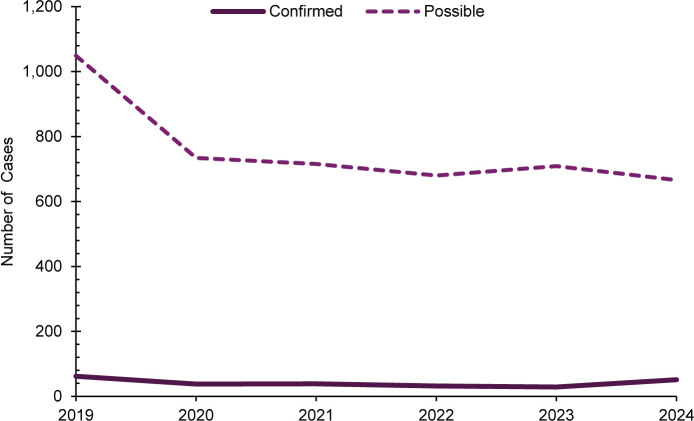
Annual Varicella Cases, All Military Health System Beneficiaries, 2019–2024

**FIGURE 8.**
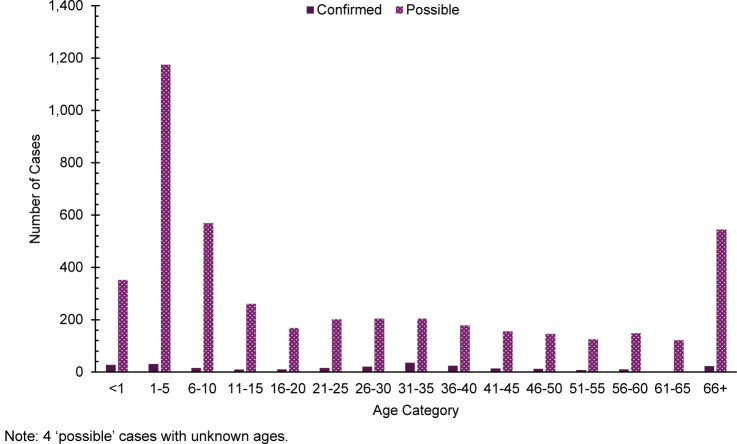
Age Distribution of Confirmed and Possible Varicella Cases, All Military Health System Beneficiaries, 2019–2024

A total of 251 confirmed and 4,554 possible varicella cases were identified among all MHS beneficiaries during the surveillance period. The majority of confirmed varicella cases (n=179, 71.3%) and possible varicella cases (n=4,071, 89.4%) were among non-service member beneficiaries **(Table 1)** . The overall trend in possible varicella cases declined by approximately 37% during the surveillance period (from 1,049 cases in 2019 to 666 cases in 2024). While the number of confirmed varicella cases remained relatively stable from 2020 through 2023, the subsequent increase to 51 confirmed cases in 2024 does not indicate a general decline over the surveillance period, as demonstrated by possible varicella case data **(Figure 7)** . Nearly 23% (n=57) of confirmed cases were among children ages 5 years and younger **(Figure 8)** . Among the 72 confirmed cases of varicella among service members, only 7 cases had been fully vaccinated, 48 cases had received an exemption from immunization, and 12 cases had not been vaccinated **(Table 2)** .

## Discussion

In this retrospective analysis from 2019 to 2024, no measles cases were identified among service members. The previous MMR/V report also demonstrated no confirmed measles cases among service members from 2016 to 2019. ^9^ For non-service member beneficiaries, measles primarily affected children ages 5 years or younger, with 50% of confirmed cases and over 57% of possible cases occurring in this age group. A similar trend was observed in the general U.S. population, with 42% of all cases among children under age 5 years in 2024. ^3^ This is especially of concern, as measles can cause serious health complications in children younger than age 5 years. ^14^ It is important to note, however, that measles continued to decrease among all MHS beneficiaries throughout the surveillance period.

During the 6-year surveillance period, there were over double the number of confirmed cases of mumps compared to measles (n=18, n=8, respectively). In the last *MSMR* report of MMR/V cases among MHS beneficiaries, confirmed mumps cases were 12 times higher than measles cases. ^9^ The increased number of mumps cases is consistent with continued mumps outbreaks across the U.S., particularly among fully vaccinated young adults. ^15^ This may be attributed to the fact that the 2-dose MMR vaccine is less effective against mumps (86%) compared to the measles (97%). ^15 - 17^ This is evident in this study, with 22% (n=4) breakthrough mumps cases that were fully vaccinated during the surveillance period. In 2017, the Advisory Committee of Immunization practices recommended a third dose of MMR (MMR3) during mumps outbreaks; and it has been proposed that MMR3 be administered in late adolescence or prior to college to help improve mumps vaccine efficacy. ^18^

Distribution of confirmed rubella cases was relatively similar in service members and non-service members. No confirmed rubella cases were among children or young adults (younger than age 20 years); most rubella cases were among adults aged 21-35 years. A larger number of possible rubella cases were identified among non-service members than service members, which may be attributed to the vaccination requirement for military service. Since rubella is no longer endemic to the U.S., cases among MHS beneficiaries were most likely acquired outside the U.S.; however, this analysis did not discern country of MMR/V acquisition.

Varicella afforded the most confirmed cases in both service members (n=72) and non-service members (n=179), and 90% (n=65) of all confirmed cases among service members were not fully vaccinated. Full vaccination against varicella among service members might decrease the number of cases among all MHS beneficiaries.

All MMR/V cases decreased from 2019 to 2020, coincident with the COVID-19 pandemic during which most people were socially distancing and taking extra hygiene precautions, such as wearing masks and frequently washing hands. The same is observed in the general U.S. population, from 1,274 cases of measles in 2019 that drastically dropped to 13 cases in 2020. There were also multiple mumps outbreaks in 2019 within the U.S. military, such as the outbreak aboard USS Fort McHenry in early 2019 and an outbreak in July 2019 among Army troopers in Italy. ^9^ Such outbreaks are contributing factors to the high number of observed cases in 2019 compared to the rest of the surveillance period. Cases of mumps and rubella started increasing, however, again in 2023 and 2022, respectively. Similar to previous reports of MMR/V among all MHS beneficiaries, ^8 , 9^ a substantially higher number of possible cases were identified than confirmed cases. Since a diagnosis of an MMR/V in this study was considered a case if reported as a confirmed RME notification, cases identified from inpatient and outpatient records that were not reported as RMEs are not counted as confirmed cases, but as possible cases. This potentially led to under-estimating confirmed MMR/V cases within the MHS.

This analysis also included MMR/V vaccination status among service members, which was not considered in previous updates. This addition is useful for determining numbers of breakthrough cases and identifying cases that were unvaccinated, providing indication of the importance of MMR/V vaccination.

The results presented may, however, be subject to data limitations. A few confirmed mumps and varicella cases among service members had no evidence of either a vaccine record or immunization exemption. It is, therefore, probable that immunization information may be missing or subject to data entry errors for some service members, as MMR/V vaccination is a requirement for military service.

Overall, the number of all MMR/V cases were higher among non-service member MHS beneficiaries compared to service members. This finding is not surprising, since evidence of immunity for MMR/V is required for service members. As MMR/V outbreaks continue to occur in the U.S. continued monitoring of MMR/V cases within the MHS is essential to ensure a healthy force and military readiness.
